# Self-Assembled Hydrogel from Pyrene-Modified Peptide as 3D Matrices for Neuronal Cell

**DOI:** 10.1021/acsabm.5c01646

**Published:** 2025-12-30

**Authors:** Devi Wahyuningtyas, Yoyo Cheng-Ting Yu, Chin-Yun Hsieh, Yung-An Huang, Ruei-Yu He, Tzu-Hung Teng, Bryan Po-Wen Chen, Jung-Ren Huang, David T. Wu, Joseph Jen-Tse Huang

**Affiliations:** † Institute of Chemistry, 38017 Academia Sinica, No. 128, Sec. 2, Academia Road, Nangang District, Taipei 11529, Taiwan; ‡ Sustainable Chemical Science and Technology, Taiwan International Graduate Program, Academia Sinica and National Taiwan University, No. 1, Sec. 4, Roosevelt Road, Daan District, Taipei 10617, Taiwan; § Department of Chemical Engineering, National Taiwan University, No. 1, Sec. 4, Roosevelt Road, Taipei 10617, Taiwan; ∥ Department of Chemistry, National Central University, No. 300, Zhong-da Road, Zhong-Li District, Taoyuan 320317, Taiwan; ⊥ Department of Chemistry, National Taiwan Normal University, No. 162, Sec. 1, Heping E. Road, Daan District, Taipei City 106, Taiwan; # Institute of Physics, Academia Sinica, No. 128, Sec. 2, Academia Road, Nangang District, Taipei 11529, Taiwan; ∇ Department of Applied Chemistry, National Chiayi University, No. 300, University Road, Chiayi 600, Taiwan

**Keywords:** pyrene, peptide hydrogel, nanofibrils, biomaterial, 3D cell culture

## Abstract

Self-assembled hydrogels offer biomimetic platforms for three-dimensional (3D) cell culture; yet, achieving stability and functionality under physiological conditions remains a challenge. Here, we report a series of pyrene (Py)-modified peptides designed to form hydrogels with tunable physical and biological properties. Among them, Py-L_3_K_3_ uniquely formed stable, fluorescent hydrogels at both neutral and basic pH, in contrast to Py-K_6_ and Py-A_3_K_3_, which gelled only at basic pH. Molecular dynamics simulations revealed pH-dependent clustering as a key mechanism driving hydrogel formation. Structural analysis showed that Py-L_3_K_3_ forms nanofibrillar networks with granular surface morphologies and β-sheet-rich conformations. Rheological studies demonstrated its solid-like viscoelastic behavior and self-healing capability, as determined by oscillatory shear measurements. Importantly, Py-L_3_K_3_ supported neuronal cell viability, attachment, and growth under physiological conditions, highlighting its potential as a 3D culture scaffold. These findings present Py-L_3_K_3_ as a promising candidate for applications in neuronal engineering and injectable regenerative biomaterials.

## Graphical abstract

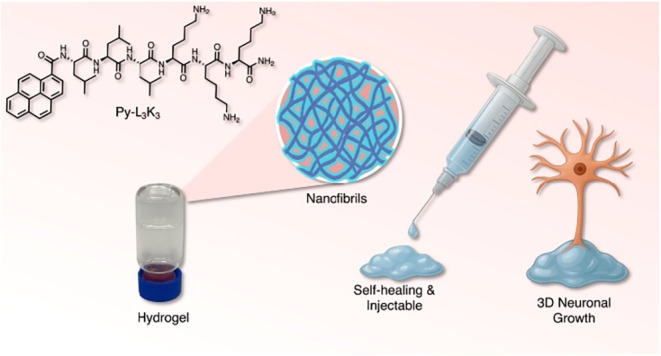

## Introduction

Hydrogels are three-dimensional polymeric networks capable of absorbing and retaining large amounts of water, resulting in flexible and soft mechanical properties similar to those of biological tissues.^1^ Their highly tunable physical and chemical characteristics have made hydrogels versatile platforms for applications across diverse fields, including agriculture, environmental remediation, soft robotics, and energy storage.^2–4^ For example, hydrogels are currently used to retain soil moisture and support plant growth under drought conditions,^5–7^ trap pollutants in water purification systems,^8–12^ and serve as functional components in soft actuators and sensors.^13–16^ In the energy sector, hydrogels are increasingly integrated into battery^17^ and supercapacitor^18^ systems. Among these broad applications, the most impactful uses of hydrogels lie in biomedicine,^19–23^ where they are applied in wound dressings,^24–26^ tissue engineering,^27–32^ biosensing,^33–35^ anticancer therapies,^36–39^ and drug delivery systems.^40–44^

Among the various classes of hydrogel-forming materials, peptide-based hydrogels have garnered significant interest due to their intrinsic biocompatibility, biodegradability, and ability to self-assemble into bioactive architectures.^45^ Compared with synthetic polymers, peptides offer greater design flexibility, allowing precise modulation of mechanical, chemical, and biological properties through rational sequence engineering or chemical modification. For biomedical applications, a key requirement is the formation of nanofibrous networks that support cellular attachment, migration, and proliferation. Porosity also plays a critical role in regulating the water content and nutrient exchange within the scaffold. Mechanically, peptide hydrogels often exhibit viscoelastic behavior, providing a combination of structural stiffness and dynamic flexibility. Their self-healing capability ensures long-term integrity in dynamic biological environments.^46^

At the molecular level, peptide hydrogels self-assemble through noncovalent interactions such as π–π stacking, electrostatic (ionic) complementarity between charged residues, and amphiphilic interactions between hydrophobic and hydrophilic amino acids.^47–50^ To enhance these interactions and promote hydrogel formation, aromatic moieties such as fluorenylmethoxycarbonyl (Fmoc),^51–53^ naphthalene (Nap),^54–57^ and naphthalene diimide (NDI)^58^ have been conjugated to peptide sequences, enabling stronger π–π and hydrophobic interactions. These self-assembled peptide hydrogels offer exciting opportunities for developing tunable materials with controlled chemical, structural, and mechanical features tailored to specific biomedical applications.

In this study, we present an integrated experimental and computational approach to develop self-assembling peptide-based hydrogels suitable for 3D cell culture. We synthesized a series of pyrene-modified peptides and screened their ability to form hydrogels under varying pH conditions. Their self-assembly behavior was characterized using biophysical and microscopic techniques, and molecular dynamics (MD) simulations were employed to uncover the pH-dependent clustering mechanisms driving gel formation. Among the candidates, Py-L_3_K_3_ formed a stable fluorescent hydrogel at neutral pH, exhibiting a nanofibrillar architecture, viscoelastic and self-healing properties, and strong biocompatibility with neuronal cells. Our findings provide a foundation for the rational design of peptide hydrogels as injectable, physiologically stable scaffolds for tissue engineering and regenerative medicine.

## Results and Discussion

### Design and Synthesis of Aromatic Hydrocarbon-Modified Peptides

To develop peptide-based self-assembling hydrogels, we synthesized a series of hexapeptides: hexalysine (K_6_), trialanine–trilysine (A_3_K_3_), and trileucine–trilysine (L_3_K_3_). Lysine, alanine, and leucine were selected for their respective contributions to electrostatic, hydrogen bonding, and hydrophobic interactions. To promote aromatic stacking and enhance self-assembly, we conjugated either pyrene (Py) or tetraphenylethylene (TPE) moieties to the N-terminus of these peptides (Figure 1A,B). Pyrene was selected as the aromaticity-promoting unit because its large, planar π-surface strengthens π–π stacking interactions and promotes ordered peptide self-assembly into nanofibrillar hydrogels. The intrinsic fluorescence of pyrene allows direct imaging under confocal or fluorescence microscopy without the need for additional dyes, facilitating a clear distinction between the hydrogel matrix and the cells. To further probe the effect of the aromatic geometry on assembly, we also prepared TPE-conjugated peptides. Although both pyrene and TPE are polycyclic aromatic hydrocarbons, pyrene is planar, whereas TPE adopts a twisted, propeller-like conformation. This structural contrast allowed us to evaluate how aromatic planarity influences fibril formation and hydrogelation. All aromatic hydrocarbon-modified peptides were synthesized using standard solid-phase peptide synthesis and purified by HPLC (see the Experimental Section for details). The identity of each peptide was confirmed by MALDI-TOF mass spectrometry, and the measured masses are listed in Table 1.

**Figure 1:**
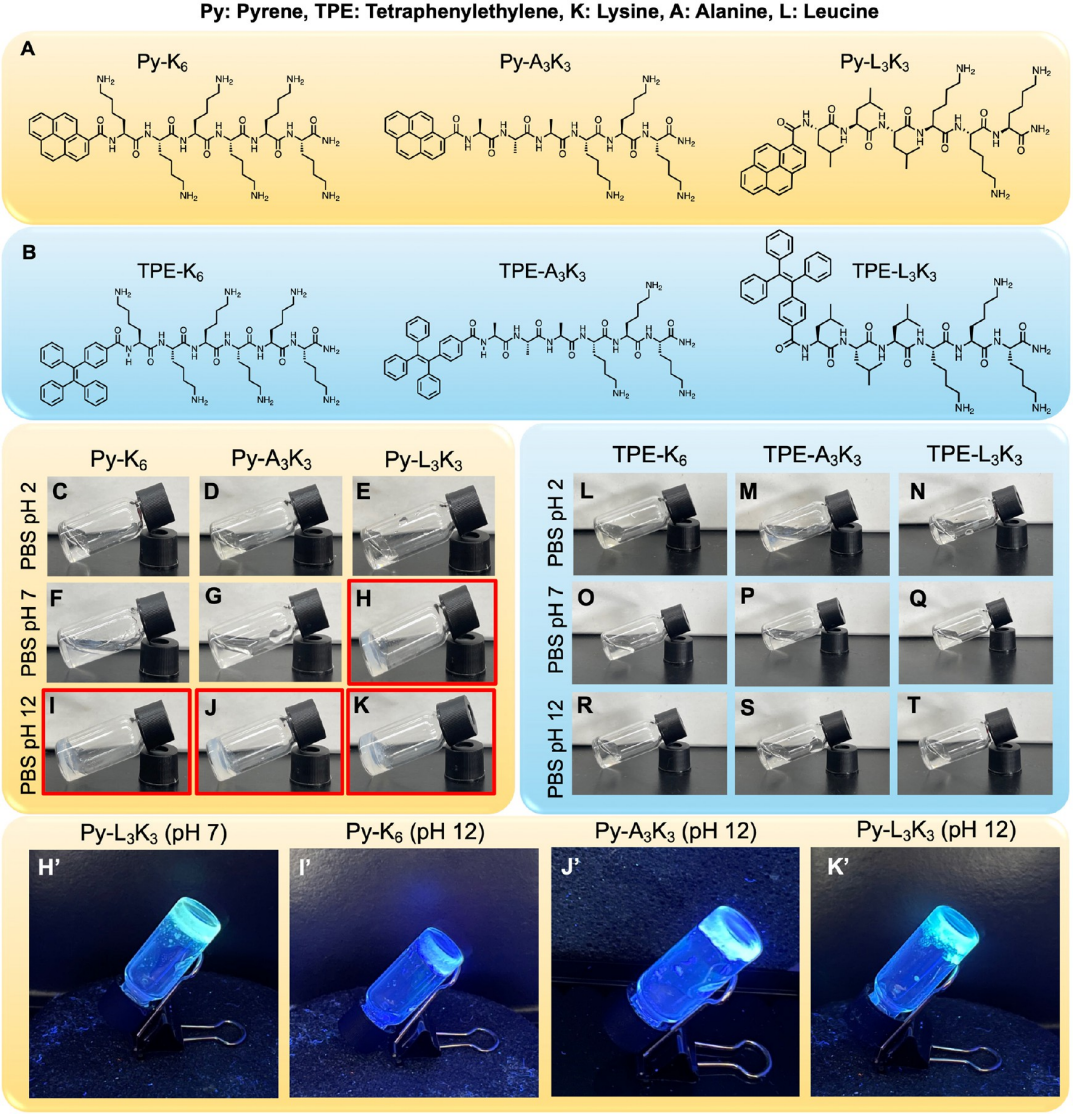
Chemical structure of (A) Py-peptides and (B) TPE-peptides. The image of the vial inversion technique to demonstrate the hydrogel formation from (C–K) Py-Peptide and (L–T) TPE-peptide at 1 wt %. The image of vial inversion technique of hydrogel from Py-peptide under UV light (H′ I′ J′ K′).

**Table 1:** List of Peptide Sequence and Their Molecular Weights

name	sequence	molecular Weight	synthetic yields
K_6_	NH_2_–KKKKKK-CONH_2_	787.07	90%
A_3_K_3_	NH_2_-AAAKKK-CONH_2_	615.80	85%
L_3_K_3_	NH_2_-LLLKKK-CONH_2_	742.02	87%
Py-K_6_	Py-KKKKKK-CONH_2_	1015.34	82%
Py-A_3_K_3_	Py-AAAKKK-CONH_2_	844.05	79%
Py-L_3_K_3_	Py-LLLKKK-CONH_2_	970.29	80%
TPE-K_6_	TPE-KKKKKK-CONH_2_	1117.52	80%
TPE-A_3_K_3_	TPE-AAAKKK-CONH_2_	972.27	79%
TPE-L_3_K_3_	TPE-LLLKKK-CONH_2_	1100.48	80%

### The Hydrogelation of Py-Modified Peptide Is pH-Dependent

To evaluate hydrogel formation, Py- and TPE-modified peptides were dissolved at 1 wt % in phosphate-buffered saline (PBS) at pH 2, 7, and 12. Gelation was assessed using the standard vial inversion test. As shown in Figure 1, Pyrene-conjugated peptides (Py-peptides) displayed strong pH-dependent gelation behavior. At acidic condition (pH 2), all Py-peptides remained soluble (Figure 1C–E), whereas at basic condition (pH 12), all formed self-supporting hydrogels (Figure 1I–K). Notably, among the three, only Py-L_3_K_3_ was able to form a hydrogel under neutral conditions (pH 7, Figure 1H), while Py-K_6_ and Py-A_3_K_3_ did not (Figure 1F,G). Owing to the pyrene moiety, all Py-peptide hydrogels exhibited fluorescence under 365 nm UV light (Figure 1H′–K′). In contrast, none of the TPE-peptides formed hydrogels under any tested pH conditions (Figure 1L–T), and their behavior was comparable to that of peptides lacking any aromatic modification (Figure S1) for the same peptide sequence and concentration. The inability of TPE-modified peptides to form hydrogels highlights that aromatic substitution alone is insufficient to drive assembly; rather, the planarity of pyrene is essential for promoting close-packed π−π stacking or cation−π stacking and fibrillar network formation. Additionally, none of the peptides, including Py- and TPE-conjugates, underwent gelation in pure water, instead remaining fully soluble (Figure S2). Together, these results establish that Py-peptides, particularly Py-L_3_K_3_, possess superior gelation ability in PBS and were therefore selected for further characterization as biofunctional hydrogels. To further investigate the optical properties of Py-L_3_K_3_ in the solution and hydrogel state, absorption and fluorescence spectra were recorded (Figure S3). At low concentrations (100 μM) in DMSO or PBS, Py-L_3_K_3_ displayed a characteristic absorption band near 340 nm (Figure S3A), attributed to the π–π* transition. In DMSO, the peptide exhibited well-resolved vibronic fluorescence peaks at 380 and 400 nm (Figure S3B). In PBS, these vibronic features were diminished, consistent with the formation of nanoscale assemblies. Upon gelation (1 wt %, PBS), the fluorescence spectrum resembled that in PBS solution and did not show excimer emission near 485 nm,^59^ suggesting that pyrene units adopt packing arrangements that restrict excimer formation within the hydrogel network.

### Molecular Dynamics Simulations Reveal pH-Dependent Self-Assembly of Py-Peptides

The structure and dynamics of self-assembled peptide-based hydrogels are governed by a range of noncovalent interactions. To better understand the pH-dependent self-assembly behavior of Py-peptides, we performed molecular dynamics (MD) simulations using the open-source GROMACS package.^60^ Details of the simulation systems are provided in Table 2. Cluster analysis was conducted using the gmx clustersize tool, which quantifies both the number and the size of clusters formed by each peptide under neutral (pH 7) and basic (pH 12) conditions. Clustering was defined as the aggregation of more than one Py-peptide molecule within a distance of 2.8 Å, as previously described.^61^ As shown in Figure 2A–C, all three Py-peptides (Py-K_6_, Py-A_3_K_3_, and Py-L_3_K_3_) tended to form a single large cluster at pH 12 (orange triangles), with a correspondingly high number of peptide molecules per cluster (Figure 2D–F, orange dots). Under neutral conditions (pH 7), distinct behaviors were observed. Only Py-L_3_K_3_ formed a single dominant cluster (Figure 2C, blue triangle; Figure 2F, blue dot), while Py-K_6_ and Py-A_3_K_3_ formed multiple smaller clusters (Figure 2A,B, blue triangles) with fewer molecules per cluster (Figure 2D,E, blue dots). Representative final simulation snapshots are shown in Figure 2G–L, highlighting the structural configurations of Py-peptide assemblies over 100 ns. Consistent with the cluster size analysis, all Py-peptides formed dense aggregates at pH 12 (Figure 2J–L). At pH 7, only Py-L_3_K_3_ showed clear aggregation into a well-defined cluster (Figure 2I), whereas Py-K_6_ and Py-A_3_K_3_ remained largely dispersed (Figure 2G,H). This trend was further supported by the average density profiles of the systems (Figure S4), which closely mirrored the clustering behavior observed in Figure 2A–C. Together, these simulation results indicate that Py-L_3_K_3_ has a higher intrinsic propensity to self-assemble into ordered aggregates under both basic and neutral conditions. This finding correlates well with its unique ability to form hydrogels at physiological pH as observed in experimental results.

**Figure 2:**
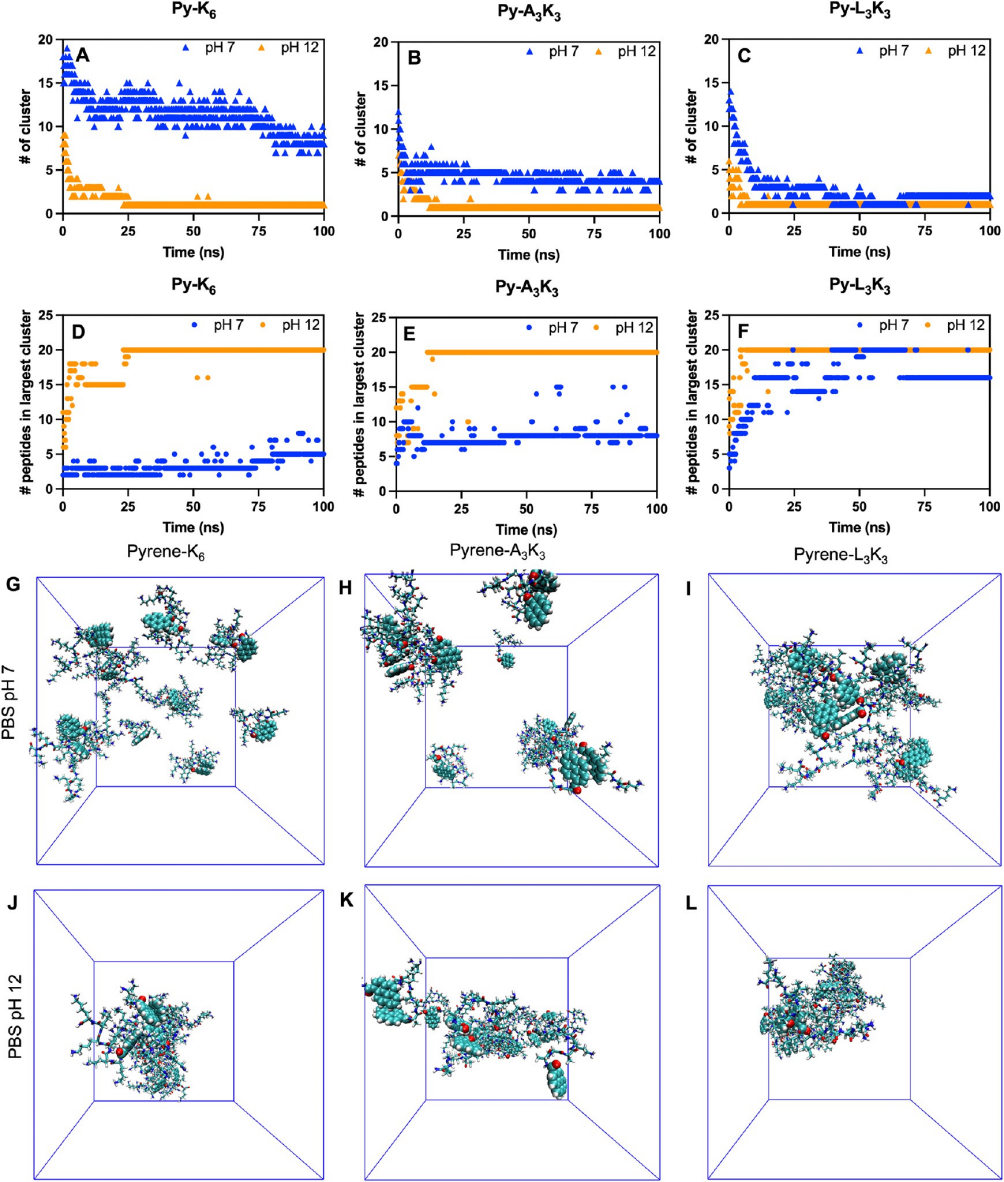
Number of cluster formation inside the simulation box along the time for (A) Py-K_6_, (B) Py-A_3_K_3_, and (C) Py-L_3_K_3_ at pH 7 (blue dot) and pH 12 (orange dot). The number of Py-peptides involved in the biggest clustering formation along the time in (D) Py-K_6_, (E) Py-A_3_K_3_, and (F) Py-L_3_K_3_ at pH 7 (blue dot) and pH 12 (orange dot). The final equilibrium state of cluster formation of Py-K_6_, Py-A_3_K_3_, and Py-L_3_K_3_ in pH 7 (G, H, I) and pH 12 (J, K, L).

**Table 2:** Detailed Compositions and the Final Aggregate States

system in Figure 2	peptide name	pH	No. of peptide molecules	volume (nm^3^)	peptide Conc. (mM)
A	Py-A_3_K_3_	7	20	365.75	91.14
B	Py-L_3_K_3_	7	20	367.25	90.76
C	Py-K_6_	7	20	364.19	91.53
D	Py-A_3_K_3_	12	20	369.21	90.28
E	Py-L_3_K_3_	12	20	369.65	90.17
F	Py-K_6_	12	20	367.95	90.59

### Characterization of Py-Peptide Hydrogel Morphology and Nanostructure

The morphological features of Py-peptide hydrogels were examined by using transmission electron microscopy (TEM). All sample was prepared at the same concentration (1 wt %). As shown in Figure 3A–D, all hydrogels formed by Py-peptides exhibited an entangled fibrillar network, consistent with self-assembled nanofiber structures. In contrast, no fibrillar structures were observed in nongel-forming samples such as Py-K_6_ and Py-A_3_K_3_ at pH 7 (Figure S5).

**Figure 3:**
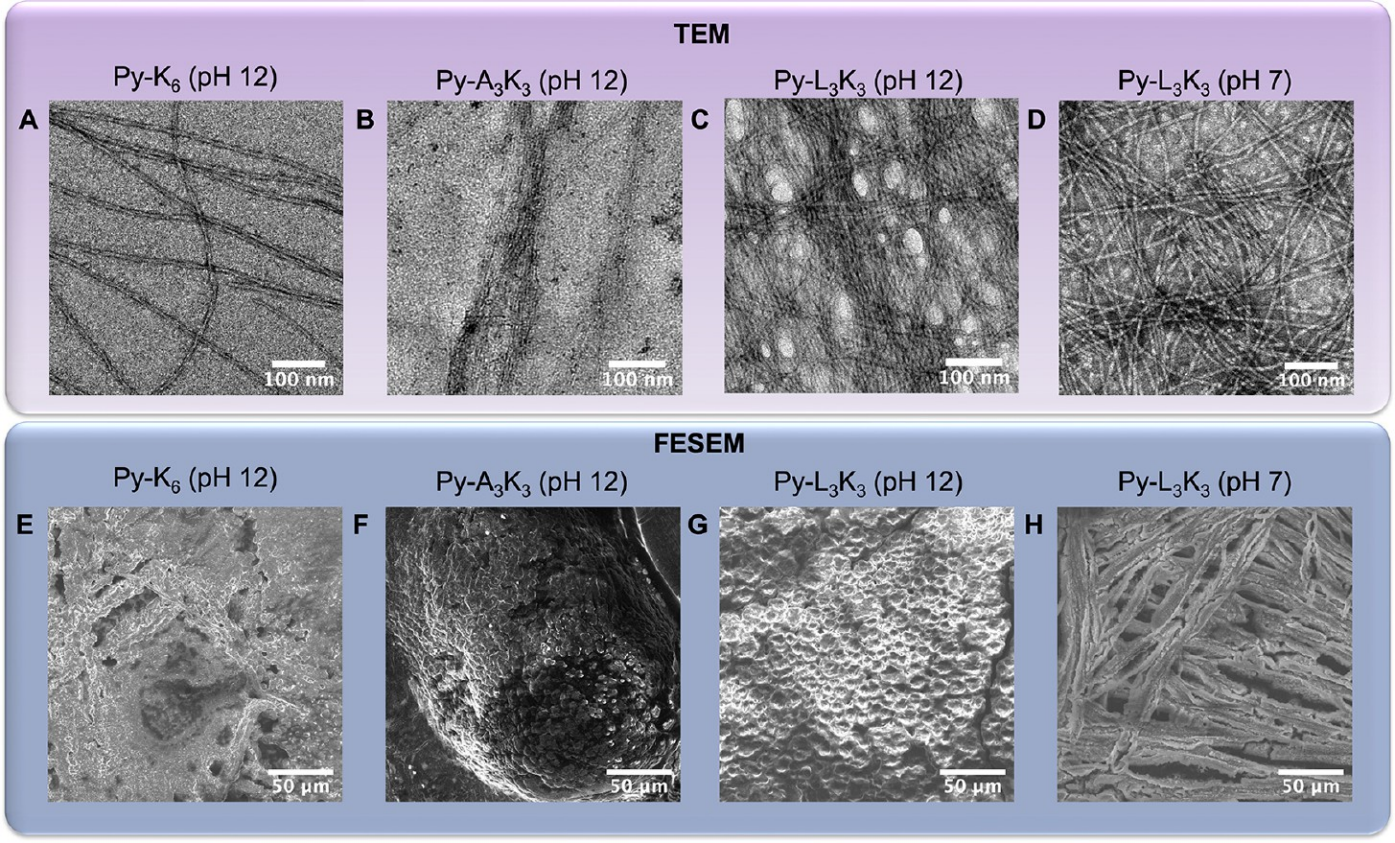
TEM images of (A) Py-K_6_, (B) Py-A_3_K_3_, (C) Py-L_3_K_3_ at pH 12, and (D) Py-L_3_K_3_ at pH 7. SEM images of (E) Py-K_6_, (F) Py-A_3_K_3_, (G) Py-L_3_K_3_ at pH 12, and (H) Py-L_3_K_3_ at pH 7. All Py-peptide samples were prepared at 1 wt % concentration.

To further investigate the secondary structural elements within these fibrillar assemblies, Fourier transform infrared (FTIR) spectroscopy was performed. As shown in Figure S6, Py-L_3_K_3_ displayed a dominant β-sheet conformation at both pH 7 and 12 (Figure S6C,F). By comparison, Py-K_6_ and Py-A_3_K_3_ exhibited a mixture of α-helix, β-sheet, and random coil structures (Figure S6A,B,D,E). These results suggest that β-sheet formation plays a key role in stabilizing the gel state of Py-peptides, particularly in Py-L_3_K_3_. Field emission scanning electron microscopy (FESEM) was also used to examine the surface morphology. At pH 12, the surfaces of Py-K_6_, Py-A_3_K_3_, and Py-L_3_K_3_ hydrogels exhibited granular textures (Figure 3E–G). Remarkably, under neutral conditions (pH 7), Py-L_3_K_3_ formed a densely packed granular network that extended into elongated fibrillar structures (Figures 3H and S7). This fibrous nanostructure is especially relevant for biomaterial applications in neuronal cell culture.^62^ Fibrillar hydrogel architectures closely mimic the structure of the brain’s extracellular matrix (ECM), providing a physiologically relevant microenvironment that supports neuronal cell adhesion, growth, and differentiation.^63^ Compared to granular architectures, fibrous scaffolds offer a higher surface-area-to-volume ratio, which enhances cell–matrix interactions.^64^ This property is essential for promoting robust neuronal cell attachment and proliferation, both of which are critical for successful 3D culture and neuronal differentiation. In summary, the nanostructural and morphological characterization confirms that Py-L_3_K_3_ forms fibrillar hydrogels under physiological conditions (pH 7), supporting its suitability as a scaffold material for neuronal cell culture applications.

### Mechanical Properties and Injectability of Py-L_3_K_3_ Hydrogel at pH 7

The mechanical properties of the Py-L_3_K_3_ hydrogel (1 wt %) at physiological pH were evaluated through rheological measurements, including time, strain, and frequency sweep, as well as three-interval thixotropy tests. These experiments were conducted to assess the hydrogel’s viscoelastic behavior, mechanical stability, and self-healing capacity by monitoring the storage modulus (*G*′) and loss modulus (*G*″) as functions of strain amplitude (γ), angular frequency (ω), and time.

As shown in Figure 4A, the time sweep (γ = 0.1%, ω = 10 rad/s) revealed gelation completion after 16 h, when both *G*′ and *G*″ reached a plateau (G′ ≈ 750 Pa and *G*″ ≈ 95 Pa). The frequency sweep data (Figure 4B, γ = 0.1%) showed that *G*′ consistently exceeded *G*″ across the tested frequency range (0.1–100 rad/s), confirming stable elastic behavior. Strain sweeps measurements (Figure 4C, ω = 10 rad/s) indicated the hydrogel maintained a viscoelastic solid state (*G*′ > *G*″) within the linear viscoelastic region (γ ≤ 1%). At higher strain amplitudes (γ ≥ 63%), *G*″ exceeded *G*′, indicating a transition to a viscoelastic fluid state. In this study, viscoelasticity refers specifically to the oscillatory rheology results, where the storage modulus remained consistently higher than the loss modulus across the tested frequency range. This reflects an elastic-dominant but still viscoelastic response, consistent with the principles of linear viscoelasticity, while clarifying that stress-relaxation tests were not performed.

**Figure 4:**
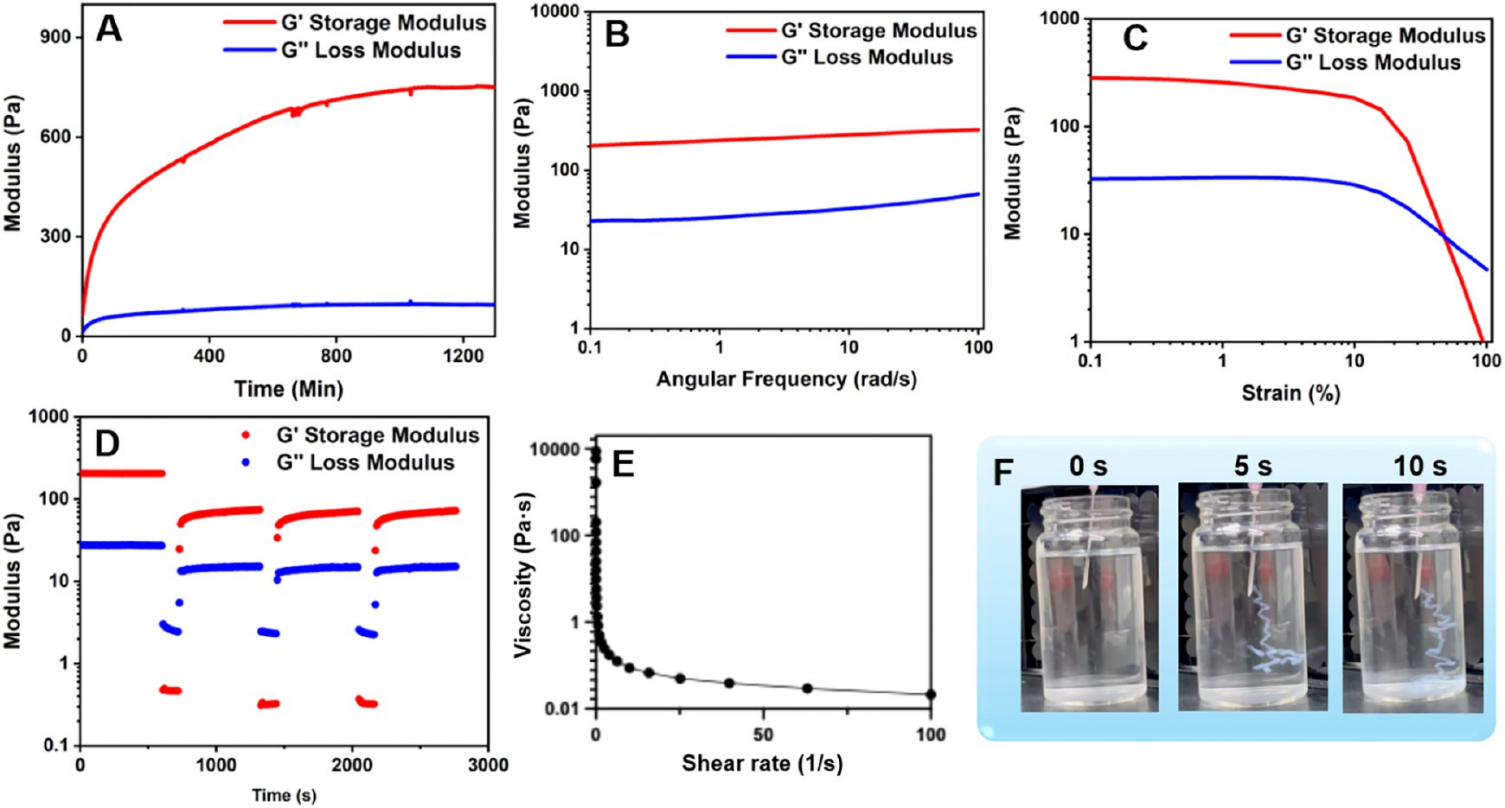
Measurement of storage modulus (*G*′) and loss modulus (*G*″) for Py-L_3_K_3_ hydrogel at γ = 0.1% and ω = 10 rad/s as a function of (A) time sweep at constant frequency and strain, (B) frequency, (C) strain, and (D) three-interval thixotropy test. (E) Viscosity of the Py-L_3_K_3_ hydrogel as a function of shear rate. (F) Snapshot of Py-L_3_K_3_ hydrogel injectability in water.

The three-interval thixotropy test (Figure 4D) further verified the hydrogel′s self-healing capacity. Under low strain (γ = 0.1%), the gel maintained its structure (*G*′ > *G*″); application of high strain (γ = 100%) temporarily disrupted the network (*G*′ < *G*″), but upon returning to low strain, both moduli recovered nearly 95% of their original values within 10 min. Repeating this cycle three times produced consistent recovery, confirming reversible network reconstruction driven by noncovalent interactions.

For comparison, the Py-L_3_K_3_ hydrogel prepared at pH 12 exhibited markedly higher stiffness and faster gelation kinetics (*G*′ ≈ 17 kPa; gelation complete after ∼7.5 h), but its network showed minimal recovery (<10% of G′ restored) after deformation (Figure S8). The enhanced rigidity at basic pH likely results from strengthened electrostatic interactions and denser peptide packing, which suppress reversible bond rearrangement and thereby hinder self-healing. Because subsequent biological experiments required a softer, self-recoverable, and physiologically relevant matrix, the pH 7 hydrogel was used for all cell-based studies.

The viscosity profile of the Py-L_3_K_3_ hydrogel at pH 7 (Figure 4E) revealed a non-Newtonian shear-thinning characteristic, where the viscosity decreased progressively with increasing shear rate. This shear-dependent behavior arises from the reversible disruption of noncovalent interactions, allowing the hydrogel to flow under applied stress and rapidly reform its network afterward. By converting the *G*′ and *G*″ values, the estimated Young’s modulus was approximately 2.3 kPa, comparable to the ultrasoft stiffness of brain and neuronal tissue (0.1–2 kPa).^65,66^ This mechanical profile supports the suitability of Py-L_3_K_3_ hydrogel for neuronal cell culture.^67^ Furthermore, extrusion tests (Figure 4F and Video S1) demonstrated smooth injection through a syringe, confirming its injectability and potential for minimally invasive delivery. Together, these findings highlight the self-recoverable viscoelasticity, shear-thinning behavior, and overall biocompatibility of the Py-L_3_K_3_ hydrogel, underscoring its promise for applications in drug delivery, tissue engineering, and regenerative medicine.

### Biocompatibility and Degradation Behavior of Py-L_3_K_3_ Hydrogel

After establishing the morphological and mechanical characteristics of the Py-L_3_K_3_ hydrogel, we next evaluated its biocompatibility and potential as a scaffold for neuronal cell culture. To assess cytocompatibility, we performed an Alamar Blue assay to measure the metabolic activity of neuroblastoma (N2a) cells incubated with hydrogel leachates for 3 days (details in Materials and Methods). The results indicated no significant reduction in cell viability, confirming that the Py-L_3_K_3_ hydrogel does not exhibit cytotoxic effects (Figure 5A). Furthermore, a comparison of N2a cell densities on Py-L_3_K_3_ hydrogel and glass substrates at days 1 and 3 showed no significant difference in proliferation (Figure 5B,C), suggesting that the hydrogel supports sustained cell growth.

**Figure 5:**
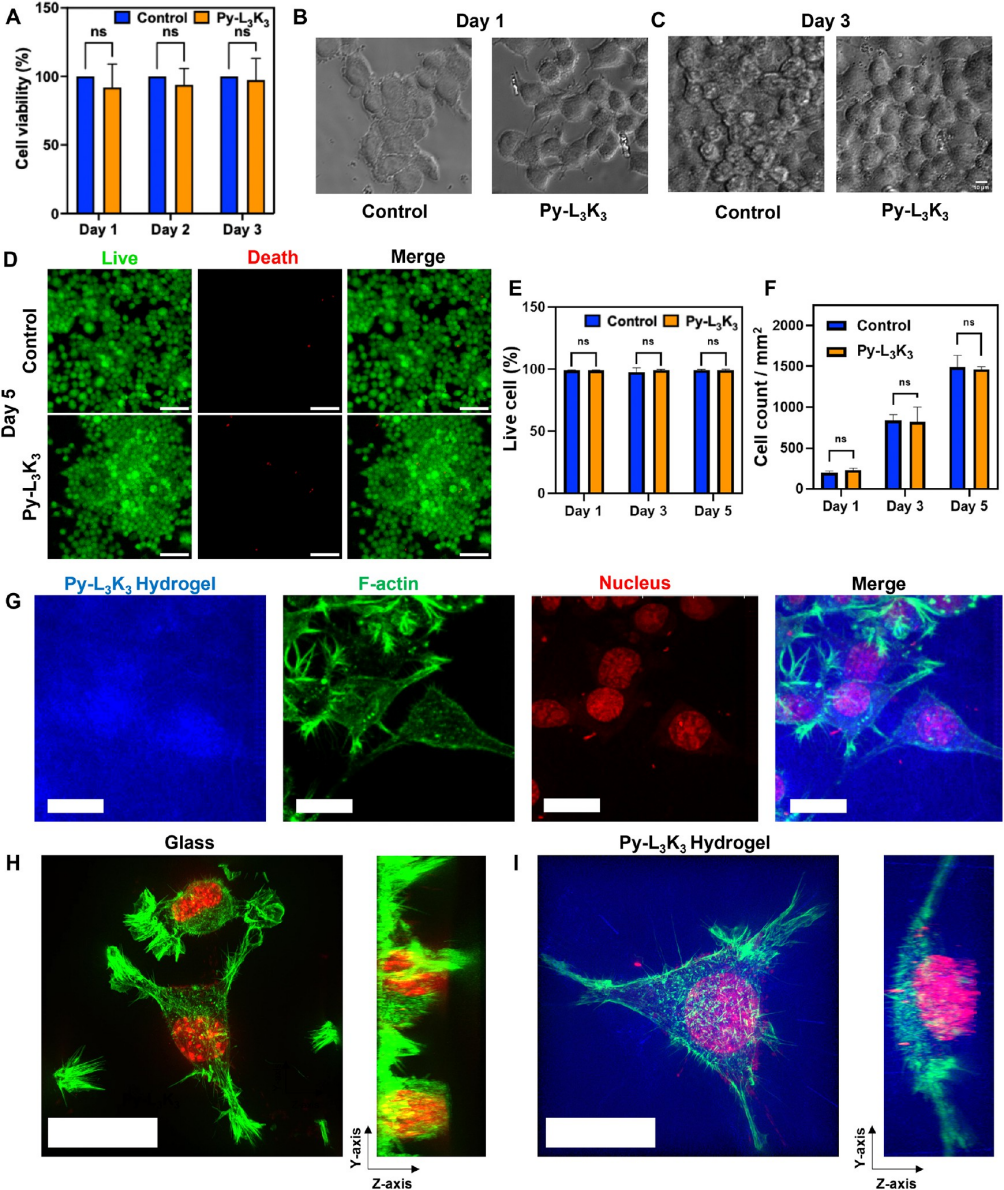
(A) Cell viability assay of Py-L_3_K_3_ hydrogel in N2a for 3 days. The bright-field image of N2a cell cultured in glass and Py-L_3_K_3_ hydrogel at (B) Day 1 and (C) Day 3. (D) Live and death fluorescence images of N2a cell cultured in glass and Py-L_3_K_3_ hydrogel for 5 days. Scale bar: 100 μm. (E, F) Quantification of (E) cell viability and (F) average cell number per 1 mm^2^ from panel (D). (G) Confocal image of N2a cell cultured in Py-L_3_K_3_ hydrogel. Super-resolution microscopy (SIM) image of N2a cell cultured in (H) glass-bottom dish and (I) Py-L_3_K_3_ hydrogel. Inset: the *Y*-*Z* axis of the 3D cell image. Scale bar: 20 μm.

To further assess long-term neuronal compatibility, N2a cells were cultured on Py-L_3_K_3_ hydrogels and glass substrates for up to 5 days and analyzed by Live/Dead fluorescence staining (Figures 5D and S9). The images showed that most cells remained viable with minimal cell death across all time points, demonstrating the hydrogel′s ability to sustain cell survival during extended culture. Quantification of the cell viability and cell count (Figure 5E,F) indicated comparable proliferation on both glass and hydrogel substrates. Together, these findings confirm that the Py-L_3_K_3_ hydrogel maintains excellent cytocompatibility and supports continuous neuronal growth over multiple days.

A Live/Dead assay using U2OS cells further demonstrated predominantly viable cells after 2 days of culture (Figure S10), supporting the finding that the Py-L_3_K_3_ hydrogel is broadly cytocompatible across different cell types. In addition, the hydrogel displayed gradual mass loss under physiological conditions (PBS, pH 7.0, 37 °C), reaching about 80% degradation after 27 days (Figure S11), indicating slow and controlled breakdown consistent with its peptide-based composition and biodegradability in aqueous environments.

### Py-L_3_K_3_ Hydrogel Supports Neuronal Growth and 3D Network Formation

To further investigate the cellular behavior, N2a cells were cultured directly on Py-L_3_K_3_ hydrogels and imaged using confocal fluorescence microscopy. The hydrogel matrix was visualized via its intrinsic blue fluorescence (excitation: 405 nm), indicating uniform hydrogel distribution and structural integrity. Cell nuclei were stained with propidium iodide, and cytoskeletal F-actin was labeled with Alexa Fluor 488-conjugated phalloidin. As shown in Figure 5G, cells cultured on Py-L_3_K_3_ hydrogels exhibited extensive F-actin-rich structures, reflecting robust cytoskeletal organization. The presence of well-distributed actin filaments and clearly defined nuclei with round to slightly elongated morphologies indicated strong cell adhesion, spreading, and viability.

Super-resolution imaging further revealed distinct morphological differences between cells cultured on glass and those within the Py-L_3_K_3_ hydrogel. As shown in Figure 5H, N2a cells infiltrated and grew within the Py-L_3_K_3_ hydrogel, forming a 3D cellular network, as illustrated by the *Z*-axis projection. The cells appeared rounder with well-distributed F-actin filaments. By contrast, cells cultured on glass substrates adopted a flattened morphology characteristic of two-dimensional environments (Figure 5I). These results highlighted that the Py-L_3_K_3_ hydrogel provides a more physiologically relevant 3D microenvironment for neuronal cell growth and organization.

To further examine neuronal organization over extended culture, N2a cells were maintained on Py-L_3_K_3_ hydrogels for 5 days and imaged by photon-counting confocal microscopy (details in Materials and Methods). As shown in Figure S12, cells extended neurite-like processes and formed interconnected networks, penetrating beyond 200 μm within the hydrogel. Immunofluorescence staining of βIII-tubulin confirmed these neuritic extensions and network-like architectures, indicating that neuronal cells established spatial organization in three dimensions. In addition, the electrical conductivity of the Py-L_3_K_3_ hydrogel was evaluated to explore its potential relevance to neuronal applications (details in the Materials and Methods). As shown in Figure S13, the hydrogel displayed a nearly linear current–voltage (*I*–*V*) response, corresponding to a conductivity of approximately 8 × 10^–8^ S·cm^–1^, which lies within the range reported for peptide-based supramolecular hydrogels.^68,69^ Although not designed as a conductive matrix, this modest electrical responsiveness may facilitate local ionic conduction and complement its biocompatible structural role in supporting neuronal cultures. Together, these results demonstrate that the Py-L_3_K_3_ hydrogel supports long-term neuronal survival, infiltration, and network formation within a physiologically relevant 3D microenvironment.

Compared with commonly used 3D matrices such as Matrigel, collagen, and alginate, Py-L_3_K_3_ hydrogels offer several distinct advantages. Matrigel, while supportive of cell growth, suffers from batch-to-batch variability and an undefined composition that complicates reproducibility.^70^ Collagen-based hydrogels promote neuronal adhesion and differentiation but often require chemical modifications to improve mechanical integrity and bioactivity.^71^ Alginate is biocompatible but lacks intrinsic cell-adhesive motifs, typically requiring functionalization with peptides like RGD to support cellular interactions.^72^ In contrast, the Py-L_3_K_3_ hydrogel is a fully synthetic, chemically defined system with tunable mechanical and biochemical properties. It enables precise control over the cell microenvironment while supporting the viability, cytoskeletal integrity, and 3D network formation. In conclusion, the Py-L_3_K_3_ hydrogel provides a stable, biocompatible, and reproducible platform for neuronal cell culture. Its ability to promote 3D cell adhesion, infiltration, and cytoskeletal organization highlights its potential as an alternative to conventional ECM-derived hydrogels for advanced tissue engineering and neurobiological applications.

## Conclusions

In summary, we successfully designed and synthesized a series of aromatic hydrocarbon-modified peptides that self-assemble into hydrogels with tunable physical and biological properties. Our results demonstrate that pyrene-modified peptides form hydrogels more effectively under basic conditions, with Py-L_3_K_3_ uniquely capable of forming a stable hydrogel at neutral pH. Molecular dynamics simulations revealed that the self-assembly behavior of these peptides is highly pH-dependent, with Py-L_3_K_3_ exhibiting a pronounced clustering tendency at both pH 7 and 12, consistent with its experimental gelation profile. Structural analyses confirmed that the hydrogels adopt entangled fibrillar networks and granular surface morphologies that support cellular attachment. Rheological measurements showed that Py-L_3_K_3_ hydrogels possess elastic, shear-thinning, and self-healing properties, suggesting their mechanical resilience and applicability in dynamic biological environments. Importantly, Py-L_3_K_3_ hydrogels exhibit negligible cytotoxicity toward N2a neuroblastoma cells and provide a supportive 3D matrix that promotes cell adhesion, proliferation, and cytoskeletal organization. Compared to traditional 2D cultures, Py-L_3_K_3_ facilitates a more physiologically relevant environment for neuronal cells and offers clear advantages over common ECM-based matrices in terms of tunability, reproducibility, and defined composition. Altogether, these findings position Py-L_3_K_3_ as a promising synthetic scaffold for the 3D neuronal culture and related biomedical applications. Its well-defined composition and favorable biofunctionality provide a strong foundation for further development in tissue engineering and regenerative medicine.

## Experimental Section

### Materials

All solutions and samples were prepared using deionized water with a resistivity of 18.2 Ω cm^–1^ from a Millipore Milli-Q water purification system. N,N-Dimethylformamide (DMF) and piperidine were purchased from Echo Chemical. Methyl *tert*-butyl ether (MTBE) was obtained from Merck. HPLC-grade acetonitrile was purchased from Fisher Chemical. N,N′-Diisopropylcarbodiimide (DIC; 99%), trifluoroacetic acid (TFA; 99%), triisopropylsilane (98%), and 1,2-ethanedithiol (EDT; 98%) were purchased from Alfa Aesar. Ethyl cyanohydroxyiminoacetate (Oxyma) was purchased from Acros Organics. 1,1,1,3,3,3-Hexafluoroisopropanol (HFIP; 99%) was purchased from Matrix Scientific. Rink Amide AM resin was purchased from Merck Millipore. All amino acids (Fmoc-Lys (Boc)–OH, Fmoc-Ala-OH, Fmoc-Leu-OH, (Fmoc: fluorenylmethyloxycarbonyl, Boc: *tert*-butyloxycarbonyl)) were purchased from AnaSpec (Fremont, USA). Phosphate-buffered saline 1X (PBS) was prepared from sodium chloride (NaCl), potassium chloride (KCl), sodium phosphate dibasic (Na_2_HPO_4_), and potassium phosphate monobasic (KH_2_PO_4_). 1-Pyrene carboxylic acid and 4-(1,2,2-triphenylethenyl) benzoic acid were purchased from Tokyo Chemical Industry (TCI).

### Peptide Synthesis, Purification, and Characterization

All peptides were synthesized through standard Fmoc polyamide chemistry on Rink amide resin by using a solid-state peptide synthesizer (Library Blue, CEM, USA). After cleavage from the resin, all crude peptides were purified by HPLC (1260 Infinity LC system, Agilent) on a C18 reversed-phase semipreparative column (Shiseido, Japan). The gradient separation was achieved by mixing buffer A (5% acetonitrile/0.1% TFA/94.9% water) and buffer B (0.1% TFA/99.9% acetonitrile). The flow rate was kept at 3 mL/min. The purified peptide was then confirmed by either matrix-assisted laser desorption/ionization (Applied Biosystem, USA) or electrospray ionization mass spectroscopy.

### Preparation of Hydrogel and Vial Inversion Method

The peptide stock was prepared by dissolving the peptide in an acetonitrile and water (1:1 ratio) mixture. Then the peptide was weighed and lyophilized to obtain the powdered sample. The hydrogel was then prepared by dissolving and mixing the peptide powder in PBS or water at room temperature. For the 1 wt % experiment, 2 mg of peptide was dissolved in 200 μL of PBS buffer. Gelation was evaluated by using the standard vial inversion method. After mixing and incubation, each sample vial was inverted upside down to assess the flow behavior. A material was classified as a hydrogel if no visible flow was observed for at least 30 s; samples that remained free-flowing were designated as solutions.

### TEM Sample Preparation and Observation

The morphology of the peptide was characterized by using a FEG-TEM (FEI Tecnai G2 TF20 Super TWIN) instrument. Peptide sample (5 μL, 1 wt %) was taken and dropped onto a copper grid (200 mesh) before being left to stand for 5 min to allow the sample to attach to the copper grid. The copper grid was dried by absorbing the solvent from the edge of the grid with filter paper. The grid was washed with water three times to remove the salt. Subsequently, staining dye (uranyl acetate, 1%, 5 μL) was dropped onto the grid and left to stand for 1 min. The staining dye was removed, and the grid was dried inside a desiccator.

### UV–Vis and Fluorescence Spectroscopy

The optical properties of Py-L_3_K_3_ were characterized in both the solution and the hydrogel state. For solution measurements, Py-L_3_K_3_ solutions (100 μM) were prepared in DMSO and PBS (pH 7.0), and UV–vis spectra were recorded from 230 to 700 nm using the corresponding solvent as reference on a V-730 spectrophotometer. Fluorescence emission spectra were collected in DMSO from 359 to 700 nm (excitation at 344 nm) and in PBS (pH 7.0) from 354 to 700 nm (excitation at 339 nm) on a FP-8350 Spectrofluorometer. For hydrogel measurements, 1 wt % Py- L_3_K_3_ hydrogels were prepared in 96-well plates using PBS (pH 7.0) and equilibrated at room temperature for 24 h. The fluorescence spectra were recorded from 354 to 700 nm under excitation at 339 nm using a TECAN Mplex multimode microplate reader.

### ATR-FTIR Measurement

The secondary structure of the peptide and polycyclic aromatic hydrocarbon-peptide hydrogel was monitored using Fourier Transform Infrared (FTIR) spectroscopy with attenuated total reflectance (ATR) mode. The peptide and polycyclic aromatic hydrocarbon-peptide were dissolved in various buffer conditions (PBS pH 2, 7, 12, and water). Then, the peptide was lyophilized for 24 h until a white, dried powder was obtained. After becoming the powder, the peptide was then measured under FTIR-ATR. The infrared spectra were then analyzed and fitted using Origin software.

### Rheological Studies

The rheological properties of Py-L_3_K_3_ were evaluated using Anton Paar (MCR301) Rheometer at 25 °C with a disposable parallel plate in 50 mm diameter and sand blasted. For the time sweep rheology measurements, Py-L_3_K_3_ peptide (30 mg) was mixed with 3 mL of PBS pH 7 to make 1 wt % hydrogel and transferred immediately to the lower plate (0.5 mm gap). The time sweep was conducted at 0.1% strain and a frequency of 10 rad/s. The moduli were recorded up to ∼20 h. The amplitude sweep measurements were conducted in the range of 0.1–100% strain with a constant frequency of 10 rad/s to estimate the linear viscoelastic range (LVR). The frequency sweep measurements were performed over a range of frequencies from 0.1 to 100 rad/s at a constant strain of 0.1%. The thixotropy measurement was performed on Py-L_3_K_3_ hydrogel by applying a shear strain of 0.1% initially for 10 min, followed by 100% strain for 2 min, and finally, the strain was released back to 0.1% and maintained for 10 min. This cycle was repeated three times at a constant angular frequency of 10 rad/s. The hydrogel formed at pH 12 was characterized under the same conditions using an Anton Paar MCR302 rheometer, except that the time sweep measurement was conducted at 0.5% strain. To examine the shear-thinning behavior, a steady-shear flow test was performed on the Py-L_3_K_3_ that formed at pH 7 hydrogel by recording viscosity over a shear rate range of 0.001–100 s^–1^ at 25 °C.

### Electrical Conductivity Measurement

The electrical conductivity of the Py-L_3_K_3_ hydrogel was measured by using a two-electrode configuration. A 1 wt % hydrogel was prepared by casting the peptide solution onto an ITO-coated glass slide and allowing it to gel overnight at room temperature. Another ITO slide was placed on top to form a sandwich-type cell. Linear sweep voltammetry (0–1 V, 0.02 V s^–1^) was conducted in PBS (pH 7.0) by using a CHI650B potentiostat. The resulting current–voltage (*I*–*V*) curves were recorded, and conductivity (σ) was determined from the slope of the linear *I*–*V* region using σ = (L/R A), where *L* = 0.05 cm and *A* = 1.674 cm^2^.

### Cell Viability Assay

The cell viability was analyzed by the Alamar Blue assay. First, we prepared a 1 wt % Py-L_3_K_3_ gel in a 35 mm dish and immersed it in growth medium (Dulbecco’s modified Eagle’s medium containing 10% fetal bovine serum supplemented with Penicillin/Streptomycin 100 units per mL) for 3 days. The gel extraction medium containing the hydrogel leachable was collected for testing the biocompatibility of the hydrogel. The N2a cells were seeded at a density of 4 × 10^3^ cells in a 96-well plate with growth medium and incubated at 37 °C under a 5% CO_2_ atmosphere for 24 h. The medium was then removed, and the cells were incubated with the hydrogel leachable (gel extraction medium) for up to 72 h. The medium was replaced with fresh growth medium containing the 10% alamar blue reagent for another 6 h. 200 μL of conditioned medium was transferred to a 96-well plate, and cell viability was determined by the increased fluorescence intensity (λ_ex_ = 560 nm, λ_em_ = 590 nm) using an Enspire multiple reader (PerkinElmer).

### Live/Dead Cell Assay

The viability on Py-L_3_K_3_ hydrogels was evaluated using a Live/Dead fluorescence assay. N2a or U2OS cells were cultured directly on preformed 1 wt % Py-L_3_K_3_ hydrogels in 35 mm dishes at 37 °C under 5% CO_2_. N2a cells were observed after 1, 3, and 5 days of culture, while U2OS cells were analyzed after 2 days. Samples were incubated with calcein-AM (2 μM) and propidium iodide (4 μM) in PBS for 30 min at room temperature. Fluorescence images were acquired by using a Nikon fluorescence microscope, where live and dead cells emitted green (λ_ex = 488 nm) and red (λ_ex = 561 nm) fluorescence, respectively.

### Py-L_3_K_3_ Hydrogel as Cell Supporting Scaffold Preparation

The Py-L_3_K_3_ hydrogel was prepared in 1 wt % concentration in a 35 mm dish. After the hydrogel was formed, it was then washed with PBS and immersed in cell medium 6–8 h prior to seeding. N2a cell line was seeded at 5 × 10^4^ cells in each well. After 3 days, the cells were fixed and stained for the nucleus (propidium iodide, Sigma P4170) and actin (Phalloidin conjugated with Alexa 488, A12379).

### *In Vitro* Degradation

The degradation behavior of the Py-L_3_K_3_ hydrogel was systematically assessed under physiological conditions (PBS, pH 7.0, and 37 °C). Briefly, 200 μL of 1 wt % hydrogel was formed at the bottom of 1.5 mL Eppendorf tubes and equilibrated for 24 h at room temperature. After removal of the supernatant, the remaining gel was weighed (*W*_0_). Fresh PBS was then added, and the samples were incubated at 37 °C for up to 27 days. At designated time intervals, the hydrogels were collected, gently blotted, and weighed (*W*_*t*_) to determine the mass retention. The degradation profile was expressed as $Degradation\left(\%\right)=(\frac{W_{t}-W_{0}}{W_{0}}\times 100\%)$ .

### Super-Resolution Structured Illumination Microscopy and Confocal Image Acquisition

Confocal laser scanning microscopy (CLSM) and super-resolution structured illumination microscopy (SIM) were used to image N2a cells cultured in Py-L_3_K_3_ hydrogel and glass-bottom dish groups. For super-resolution SIM imaging, a 100X NA 1.40 Plan Apochromat objective was used to achieve enhanced spatial resolution. Cells were imaged in both glass-bottom dishes and Py-L_3_K_3_ hydrogel to compare morphological differences. Structured illumination was applied using 405, 488, 561, and 642 nm lasers, and fluorescence emission was detected with an Andor EM-CCD camera (iXon DU897). This approach allowed for high-contrast visualization of cellular structures, providing detailed insights into the cytoskeletal organization and subcellular morphology in different culture environments. For confocal imaging, a ZEISS LSM 980 system equipped with a 63*X*/1.4 NA Plan Apochromat objective and two photomultiplier tube (PMT) detectors was used. Fluorescence excitation was performed with 405, 488, 561, and 642 nm lasers, and images were acquired using ZEN 3.2 (Blue edition) software. For live-cell imaging, a top-stage incubator was used to maintain stable temperature, humidity, and CO_2_ levels, enabling time-lapse imaging of cells in hydrogel matrices. In addition, three-dimensional photon-counting confocal imaging of N2a cells cultured within Py-L_3_K_3_ hydrogels was performed using an Evident FV4000 confocal microscope equipped with a 25× silicone oil immersion objective (NA = 0.85, working distance = 1 mm). Z-stack images were collected at 1 μm intervals across depths exceeding 200 μm to visualize the spatial organization and network formation of neuronal cells within the hydrogel matrix. The photon-counting detection mode enhanced the signal-to-noise performance, enabling high-sensitivity reconstruction of 3D cellular networks.

### FE SEM Sample Preparation and Observation

FE-SEM images of Py-L_3_K_3_ hydrogel (1 wt %) were taken in a lyophilized sample and a graphene-covered sample using an Ultra Plus–Carl Zeiss instrument. For the lyophilized hydrogel, a small amount of hydrogel was placed on carbon tape and freeze-dried overnight. Then, the samples were platinum-sputtered before observation. The FE-SEM images were captured at an operating voltage of 10 keV. For the graphene-covered sample observation, the hydrogel was dropped on the surface of silicon nitride (SiN). CVD graphene on copper foil pretreated with PMMA was purchased from ACS Materials. The CVD graphene on copper foil was cut into pieces to cover the whole hydrogel. Before that, the copper was removed by soaking the foil cut in 50 mL of 0.2 M Na_2_S_2_O_8_ for 4 h. The graphene-PMMA stack was transferred to a fresh water bath, so it floated on the water surface. To cover the hydrogel with graphene, the SiN wafer containing the hydrogel was used to scoop up the graphene-PMMA stack floating on water. The stack was allowed to adhere to the sample for 10 min in the air. To remove PMMA, the sample was dipped in acetone for 2 min and rinsed off briefly in isopropyl alcohol.

### Molecular Dynamics Simulation

We conducted NPT molecular dynamics simulations using the open-source GROMACS-2022.1 package, with a 1 fs time step and a 1 ps coupling time constant for the Nosé-Hoover thermostat^73^ and a 2 ps coupling time constant for the Berendsen barostat^74^ for NPT preequilibration steps and a 2 ps coupling time constant for the Parrinello–Rahman barostat^75^ for production steps. The four-site TIP4P-2005 model was used for water, and the CHARMM force field^76^ was used for Py-A_3_K_3_-3NH_2_, Py-L_3_K_3_-3NH_2_, Py-K_6_-6NH_2_, Py-A_3_K_3_-3NH_3_^+^, Py-L_3_K_3_-3NH_3_^+^, Py-K_6_-6NH_3_^+^, and Cl^–^ ions. All the peptide structures were from CHARMM-GUI.^77,78^ Then we utilized gmx insert-molecules to put 20 peptides into a 7.2 × 7.2 × 7.2 nm^3^ cubic box and used gmx solvate to fill the box with water molecules. For pH 7 cases, we used gmx genions to neutralize the system by adding Cl^–^ ions to the box. The simulations were first initialized by energy minimization using a steepest descent algorithm followed by a 500 ps NVT preequilibration at 300 K. Second, we performed a 500 ps NPT preequilibration at 300 K and 1 bar. Finally, equilibrated measurements were taken during a 100 ns NPT run at 300 K and 1 bar. After performing equilibrated measurement, we performed gmx clustsize to measure the Py-peptides cluster size by measuring how many Py-peptides will attach each other within 2.8 Å. This comprehensive approach allows for a detailed exploration of the interactions within the system, providing valuable insights into the behavior of peptide molecules to form clusters.

## Supplementary Material





## Notes

Additional experimental methods and characterization data; peptide synthesis and purification details; UV–Vis and fluorescence spectra of Py-L_3_K_3_ in solution and gel states; TEM and SEM images; FTIR spectra; rheological measurements (time, frequency, strain sweeps, and thixotropy); molecular dynamics simulation results; Live/Dead assay data for N2a and U2OS cells; long-term hydrogel degradation profiles; 3D confocal imaging of neurite network formation; and and electrical conductivity measurements (DOCX)
Extrusion tests (MP4)

## Abbreviations

FMOC: fluorenyl methoxycarbonyl
Nap: naphthalene
NDI: naphthalene diimide
FE-SEM: field emission scanning electron microscopy
MD: molecular dynamic
TPE: tetraphenylethylene
Py: Pyrene
HPLC: high-performance liquid chromatography
TEM: transmission electron microscopy
MALDI-TOF: matrix-assisted laser desorption/ionization-time-of-flight
PBS: Phosphate-Buffered Saline
FTIR: Fourier transform infrared
3iTT: three-interval thixotropy test
