# Supplementary material for: Self-Assembled Hydrogel from Pyrene-Modified Peptide as 3D Matrices for Neuronal Cell
Source: ACS Appl Bio Mater. 2025 Dec 30;9(2):800–12. doi: 10.1021/acsabm.5c01646 (PMC12820978; doi:10.1021/acsabm.5c01646)
Supplement: Supplementary file 1 [file mt5c01646_si_001.docx]

**Supporting Information**

Self-assembled hydrogel from pyrene-modified peptide as 3D matrices for neuronal cell

*Devi Wahyuningtyas,^1^ Yoyo Cheng-Ting Yu^1,2,3^, Chin-Yun Hsieh,^1,4^ Yung-An Huang,^1^, Ruei-Yu He,^1^ Tzu-Hung Teng,^1,5^ Bryan Po-Wen Chen ^1^, Jung-Ren Huang,^6^ David T. Wu,^1,3^ Joseph Jen-Tse Huang*^1,7^*

^1^Institute of Chemistry, Academia Sinica, No. 128, Sec. 2, Academia Road, Nangang District, Taipei 11529, Taiwan.

^2^Sustainable Chemical Science and Technology, Taiwan International Graduate Program, Academia Sinica and National Taiwan University, No. 1, Sec. 4, Roosevelt Road, Daan District, Taipei 10617, Taiwan.

^3^Department of Chemical Engineering, National Taiwan University, No. 1, Sec. 4, Roosevelt Road, Taipei 10617, Taiwan.

^4^Department of Chemistry, National Central University, No. 300, Zhong-da Road, Zhong-Li District, Taoyuan 320317, Taiwan.

^5^Department of Chemistry, National Taiwan Normal University, No. 162, Sec. 1, Heping E. Road, Daan District, Taipei City 106, Taiwan.

^6^Institute of Physics, Academia Sinica, No. 128, Sec. 2, Academia Road, Nangang District, Taipei 11529, Taiwan.

^7^Department of Applied Chemistry, National Chiayi University, No. 300, University Road, Chiayi 600, Taiwan.

^*^Corresponding author: [jthuang@as.edu.tw](mailto:jthuang@as.edu.tw)

KEYWORDS. Pyrene, Peptide Hydrogel, Nanofibrils, Biomaterial, 3D cell culture.


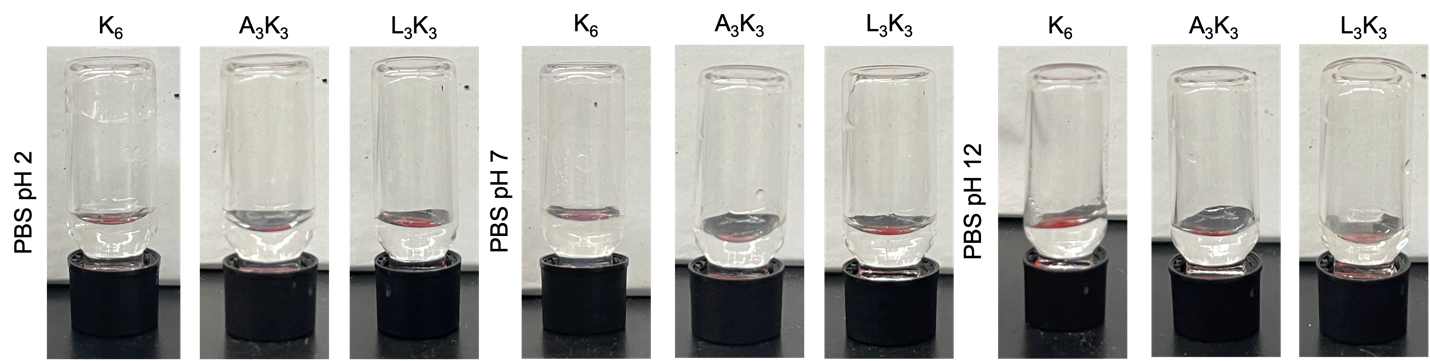


Figure S1. The image from inversion bottle technique of control peptide in different pH condition.


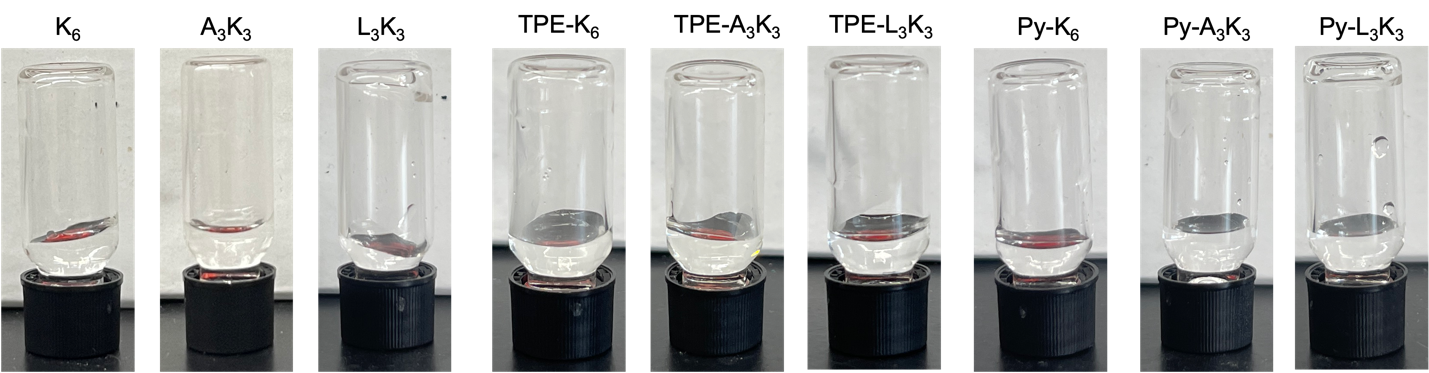


Figure S2. The image of vial inversion technique from peptides, TPE-modified peptides, and pyrene-modified peptides in water.


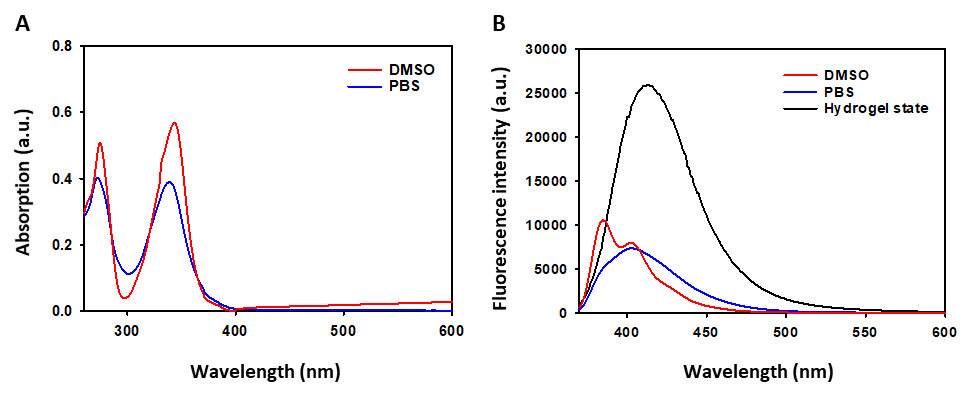


Figure S3. (A) UV–Vis absorption spectra of Py-L_3_K_3_ (100 μM) in solution state (DMSO and PBS). (B) Fluorescence spectra of Py-L_3_K_3_ dissolved in DMSO, PBS (100 μM) and in the hydrogel state (1 wt%).


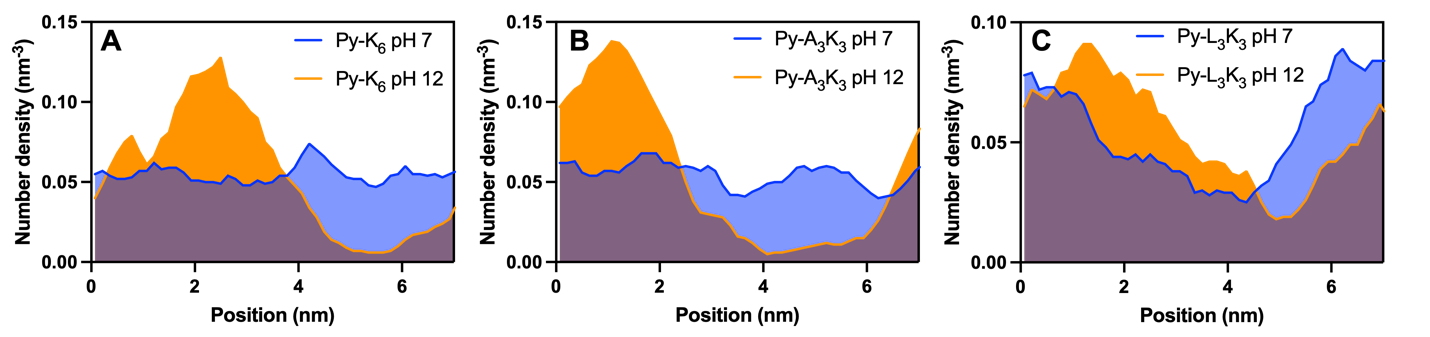


Figure S4. The number of clusters formation during the time of molecular dynamic simulation for (A) Py-K_6_, (B) Py-A_3_K_3_, and (C) Py-L_3_K_3_.


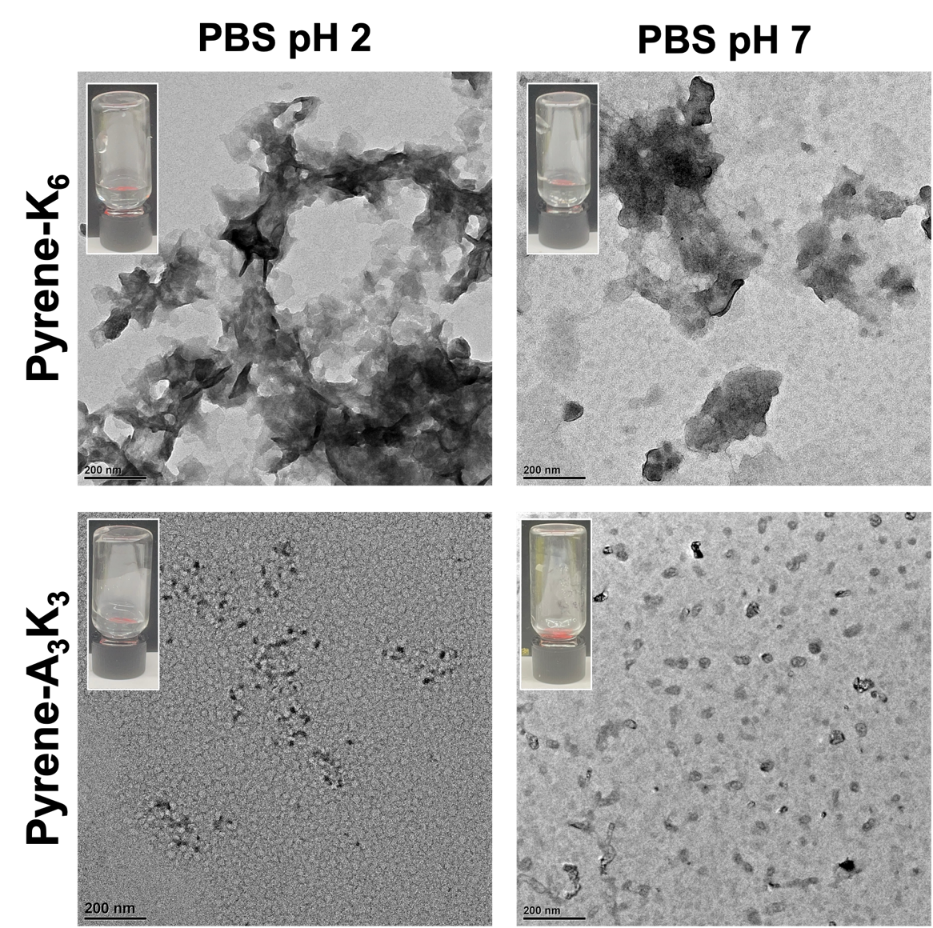


Figure S5. The TEM images of Py-A_3_K_3_ and Py-K_6_ in PBS pH 2 and pH 7.

Figure S6. The FTIR spectroscopy of pyrene-modified peptides at different pH. (A–C) FTIR spectra of Py-K_6_, Py-A_3_K_3_, and Py-L_3_K_3_ measured at pH 7. (D-F) FTIR spectra of Py-K_6_, Py-A_3_K_3_, and Py-L_3_K_3_ measured at pH 12.


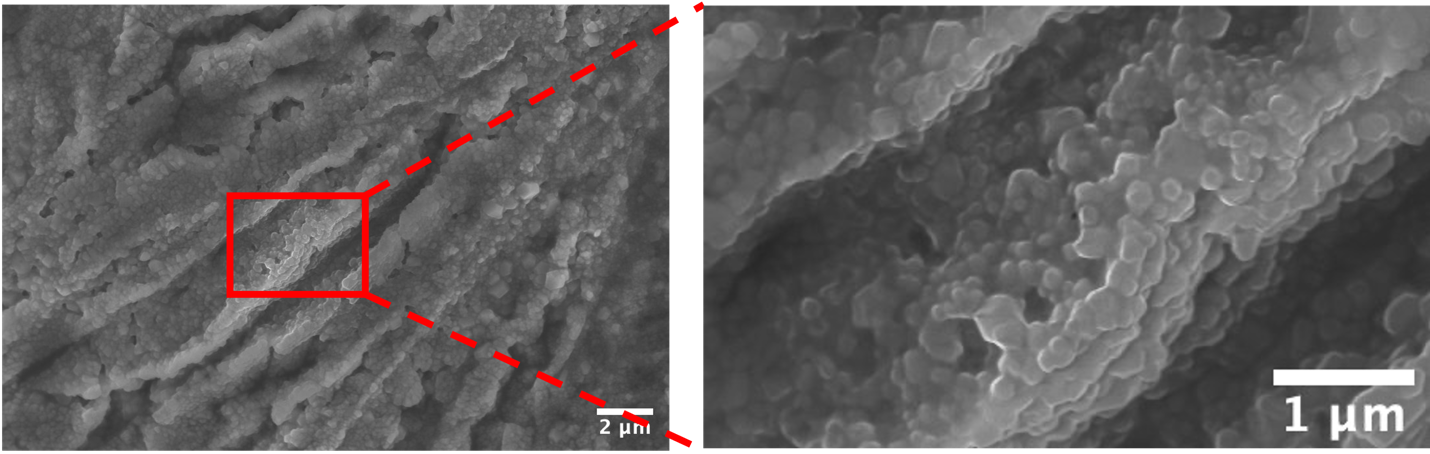


Figure S7. SEM of Py-L_3_K_3_ in PBS pH 7 prepared by lyophilized method.


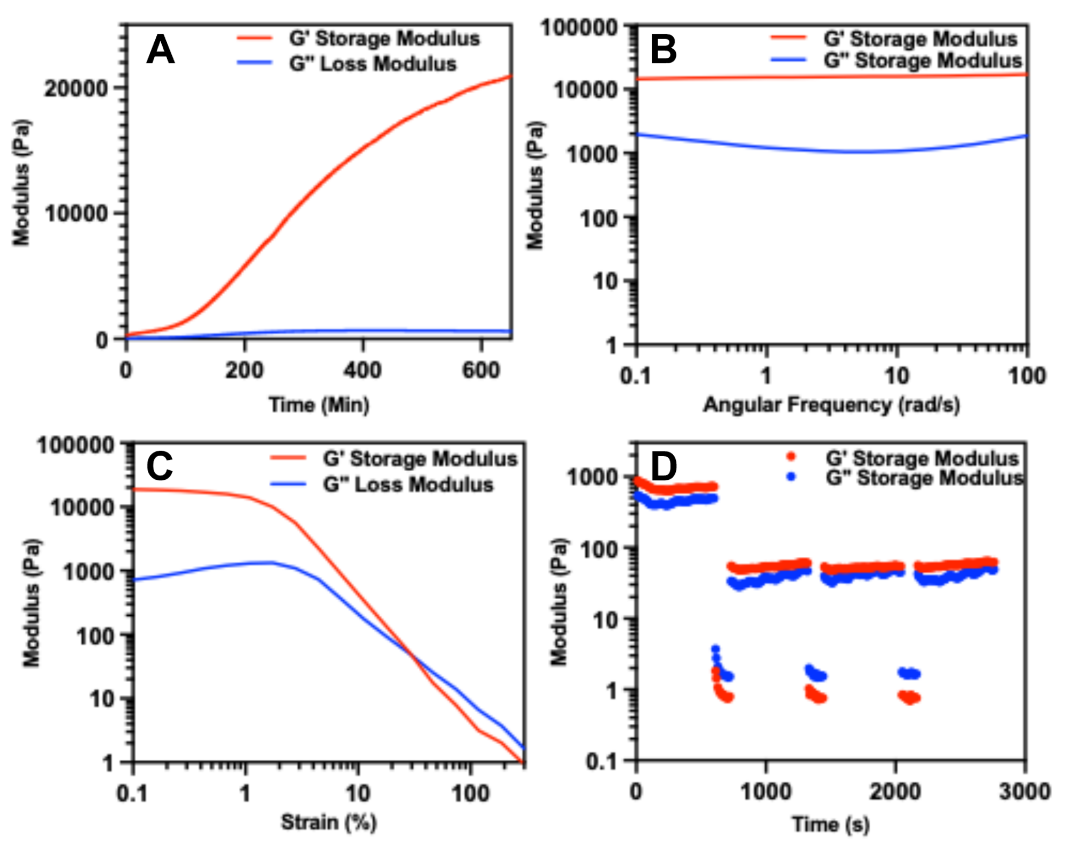


Figure S8. Measurement of storage modulus (G′) and loss modulus (G″) of the Py-L_3_K_3_ hydrogel formed at pH 12 at γ = 0.5% and ω = 10 rad s⁻¹, shown as a function of (A) time (time-sweep at constant frequency and strain), (B) frequency, (C) strain, and (D) a three-interval thixotropy test.


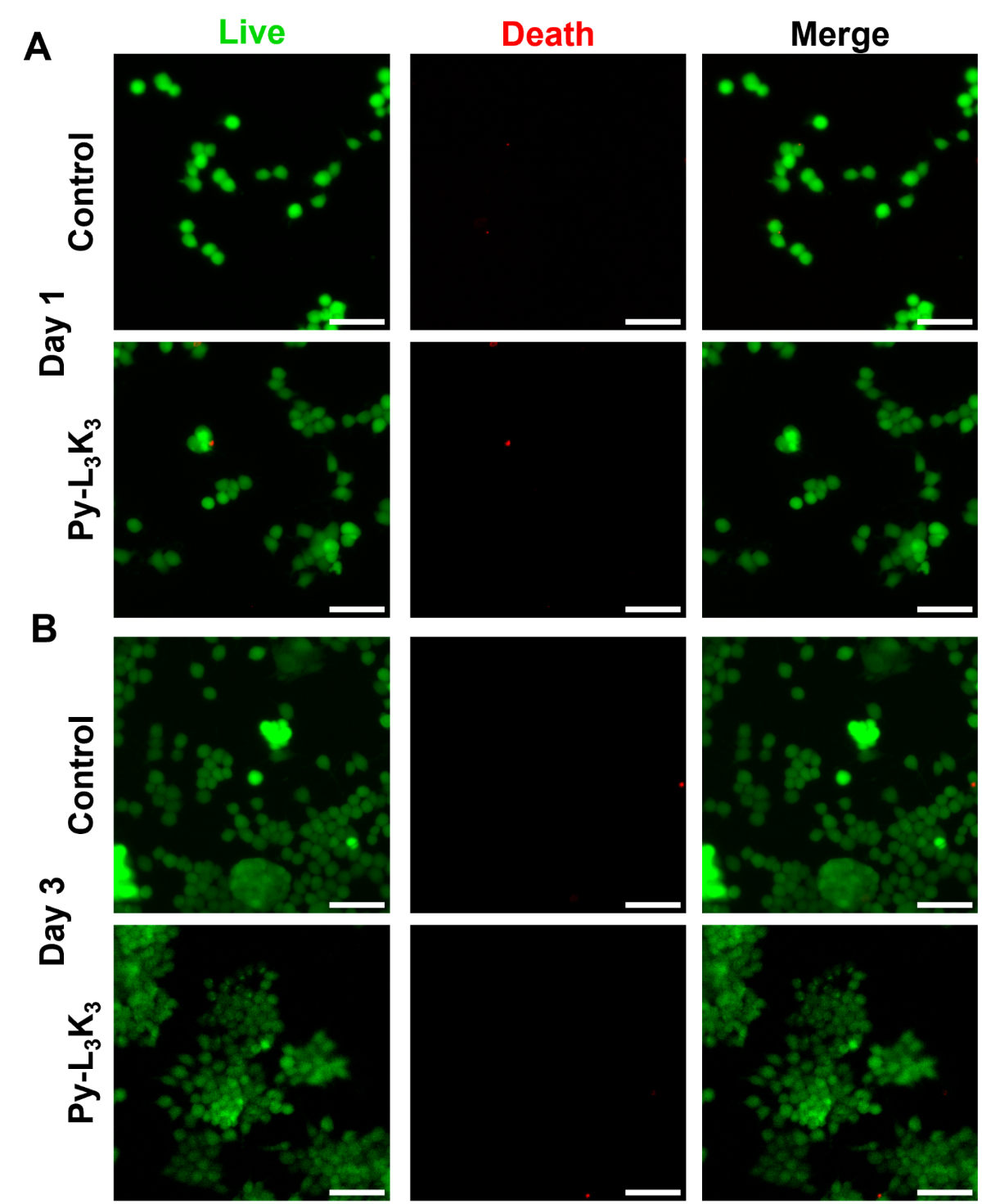


Figure S9. Live/Dead fluorescence images of N2a cells cultured on glass and Py-L_3_K_3_ hydrogel for (A) 1 day and (B) 3 days. Most cells exhibited strong green fluorescence (calcein AM, live) with minimal red fluorescence (ethidium homodimer-1, dead), indicating high cell viability on both substrates. Scale bar: 100 μm.


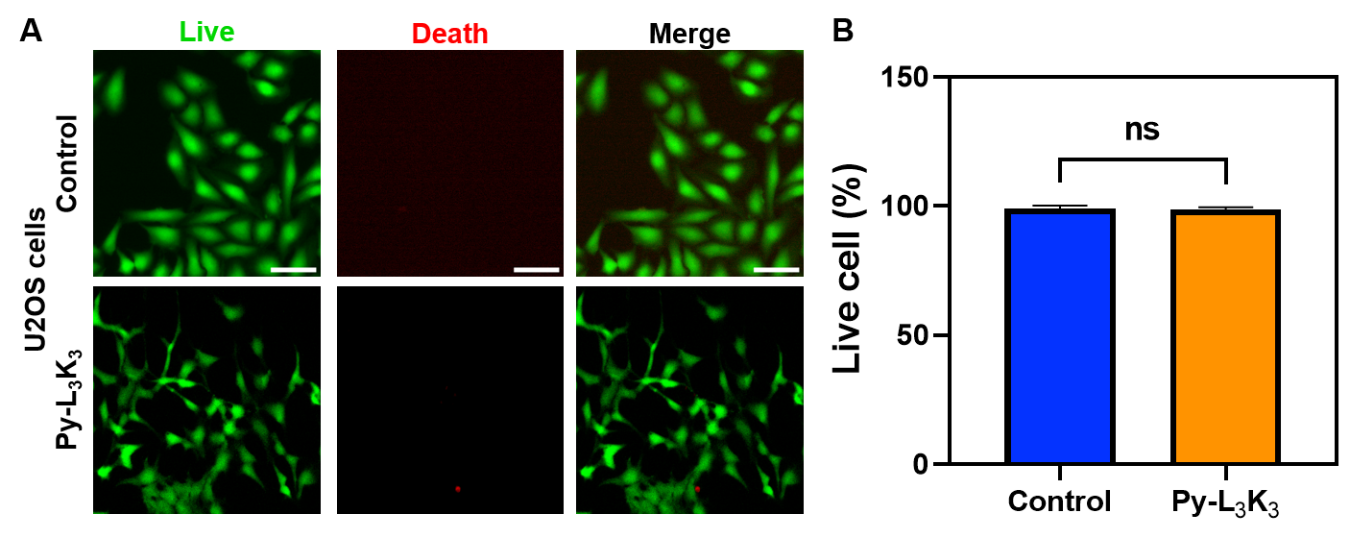


Figure S10. (A) Live/Dead fluorescence images of U2OS cells cultured on Py-L_3_K_3_ hydrogel or glass for 2 days. (B) Quantification of cell viability showing a high live cell ratio, indicating that the Py-L₃K₃ hydrogel is biocompatible. Scale bar: 100 µm.


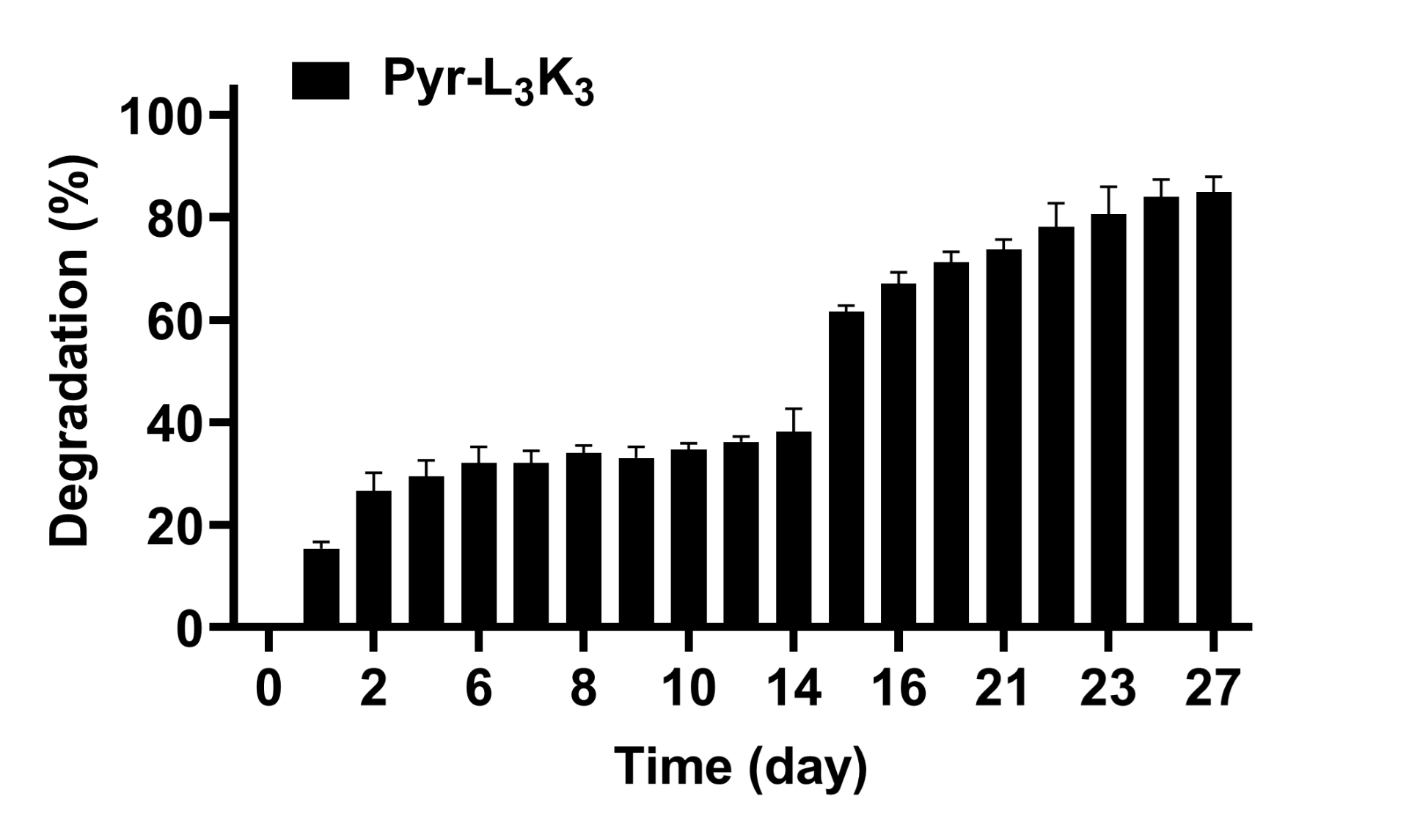


Figure S11. The degradation behavior of the Py-L_3_K_3_ hydrogel under physiological conditions (PBS, pH 7.0, 37 °C)


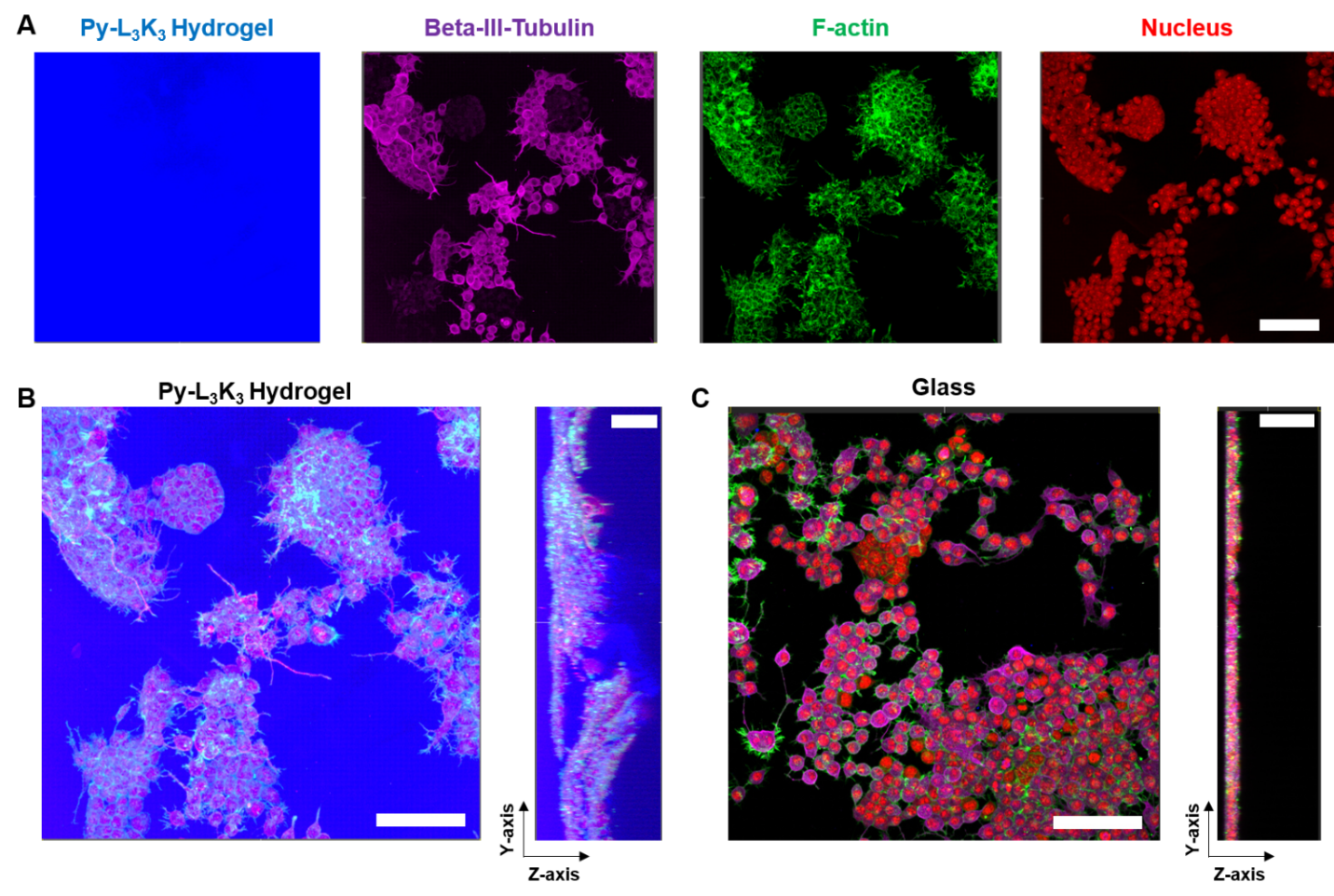


Figure S12. (A) Confocal fluorescence images of N2a cells cultured in the Py-L_3_K_3_ hydrogel for 5 days. (B) Merge images and Y–Z cross-sectional view showing cell distribution beyond 200 μm in depth. (C) Confocal image of cells on glass for 5 days with the corresponding Y–Z view. Scale bars: 100 μm.


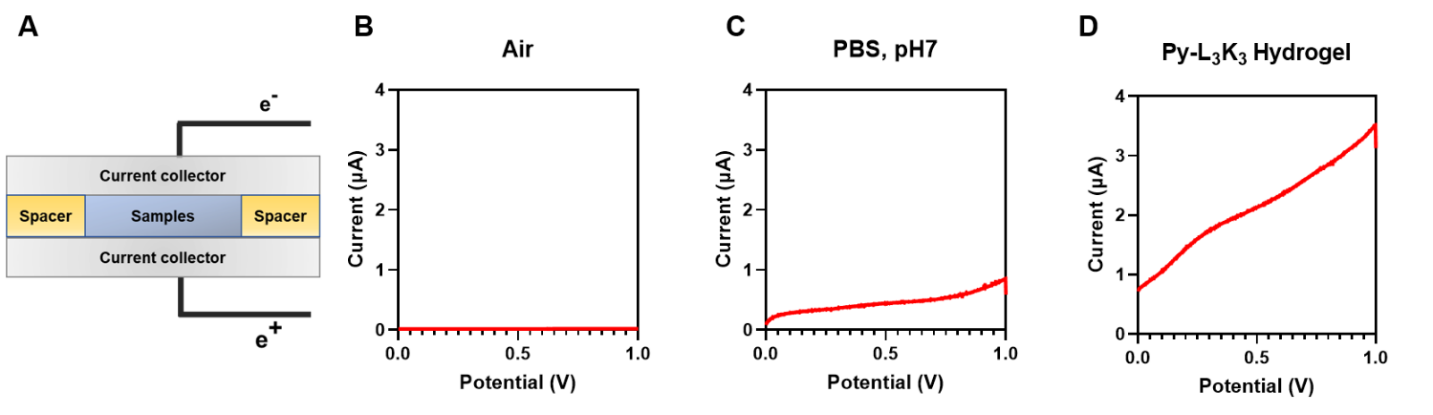


Figure S13. Current measurement of Py-L_3_K_3_ hydrogel using a two-electrode setup: (A) schematic illustration; (B) Air (blank control); (C) PBS, pH7 (buffer control); (D) Py-L_3_K_3_ hydrogel. The hydrogel displayed a weak but measurable current response, corresponding to an estimated conductivity of approximately 8.2 × 10⁻⁸ S cm⁻¹ under these conditions.
